# Adaptive Group Coordination and Role Differentiation

**DOI:** 10.1371/journal.pone.0022377

**Published:** 2011-07-19

**Authors:** Michael E. Roberts, Robert L. Goldstone

**Affiliations:** 1 Department of Psychology, DePauw University, Greencastle, Indiana, United States of America; 2 Psychological and Brain Sciences Department, Indiana University, Bloomington, Indiana, United States of America; Cajal Institute, Consejo Superior de Investigaciones Científicas, Spain

## Abstract

Many real world situations (potluck dinners, academic departments, sports teams, corporate divisions, committees, seminar classes, etc.) involve actors adjusting their contributions in order to achieve a mutually satisfactory group goal, a win-win result. However, the majority of human group research has involved situations where groups perform poorly because task constraints promote either individual maximization behavior or diffusion of responsibility, and even successful tasks generally involve the propagation of one correct solution through a group. Here we introduce a group task that requires complementary actions among participants in order to reach a shared goal. Without communication, group members submit numbers in an attempt to collectively sum to a randomly selected target number. After receiving group feedback, members adjust their submitted numbers until the target number is reached. For all groups, performance improves with task experience, and group reactivity decreases over rounds. Our empirical results provide evidence for adaptive coordination in human groups, and as the coordination costs increase with group size, large groups adapt through spontaneous role differentiation and self-consistency among members. We suggest several agent-based models with different rules for agent reactions, and we show that the empirical results are best fit by a flexible, adaptive agent strategy in which agents decrease their reactions when the group feedback changes. The task offers a simple experimental platform for studying the general problem of group coordination while maximizing group returns, and we distinguish the task from several games in behavioral game theory.

## Introduction

Groups often struggle to balance incentives for individual members with incentives for the collective group. In standard common pool resource problems [1], [2], individuals are tempted to maximize individual resources, but this behavior destroys the resource and ultimately harms everyone. Conflicting incentives in real world situations such as pollution and harvesting of natural resources understandably stretch a group's ability to coordinate for the common good, but it is still unclear how group members coordinate their actions in more constrained situations where only a shared goal exists.

Humans routinely form groups to achieve goals that no individual can accomplish alone, and presumably groups must flexibly and adaptively coordinate members' efforts in order to achieve shared goals. For example, research labs rely on the combined contributions of individuals to develop a research program and lab reputation that leads to grant funding, which may in turn benefit all of the lab's researchers. Similarly, statistical analyses in baseball and basketball increasingly value players based on the team's performance while the player is in the game, rather than individual statistics such as points scored [3].

Empirical studies from social psychology and economics have shown that group members can adequately share pieces of information under the right circumstances [4], [5], and some group learning can occur via indirect feedback [6], but it is still unclear how a division of labor develops to achieve a group goal. For example, a potluck dinner ideally coordinates participants' food contributions so there is enough to sate everyone, without excess left-overs that no one wants to take home. However, individuals often make unilateral decisions about how much food to bring to the potluck. The question then arises of how the group as a whole can coordinate the correct amount of food to bring, with some individuals volunteering to bring extra food to make up for other individuals who forget to bring any food. Most readers will recognize a similar form of coordination in committee meetings and seminars. These situations have group goals of balanced discussions and reasonable conclusions on a topic. Although each member could probably propose many ideas for or against a given topic, such exhaustive treatment is often unnecessary and even counter-productive. Ideally, the shared goals are not sullied by individual incentives, and people are often willing to cede the floor if they believe the group can consequently reach an appropriate decision with less strife or less effort. Importantly, such inactivity need not be viewed as passivity or diffusion of responsibility, because it can actually be a calculated decision to facilitate group performance.

In order to isolate and test the coordination capacities of groups, we developed a simple round-based group game called “Group Binary Search” (GBS) that creates a test bed for pure coordination without competing individual goals. In the GBS game, a computer server randomly chooses a number between 51 and 100, and without communication, each group member submits a guess between 0 and 50. The computer compares the sum of participants' numbers to its selected number, and broadcasts the same directional (e.g. “Too High”) or numeric (e.g. “Too Low by 17”) feedback to all members. During the next round, members can adjust their guesses and receive the new feedback, and the game continues until the group correctly sums to the computer's number. We coined the name Group Binary Search after the binary search algorithm in computer science [7], which searches for a number in a sorted list by iteratively guessing the median number in the current range of possibilities. An individual optimal searcher can solve a binary search task in an average of log_2_(N)-1 trials. In our task, the answer can vary from 51 to 100, so an individual's average rounds to solution would be 4.64. However, in a group binary search task, members must coordinate to reach the shared goal.

Our GBS game shares qualities of several other tasks from game theory and behavioral economics, but GBS uniquely tests participants' adaptive coordination strategies when only a shared group goal exists. The most important distinctions include the fact that achieving the shared group goal is also the only individual incentive, the goal is unknown, so participants must rely on group feedback to adjust their guesses, and complementary actions can combine to reach the group goal. Cooperative games such as Prisoner's Dilemma – and more generally, public goods games – can lead to mutual reciprocity, and coordination games such as Battle of the Sexes [8], Leader, and the route choice game can lead to alternating reciprocity [9], [10]. In order-statistic games (the general class that encompasses stag-hunt and weak-link games) [8], [11], each person's payoff is affected by the minimum valued action chosen by any member. Order-statistic games offer opposing incentives between a payoff dominant equilibrium (if everyone coordinates to the maximum valued action) and a risk dominant equilibrium (individuals choose the minimum action, which provides a decent payoff without penalizing them for others' choices). Continental Divide games [8] reward individuals for their guesses in relation to the median of everyone's guesses, and a divide separates the sub-optimal side (e.g., median guesses between 1–7 may lead individuals to a sub-optimal equilibrium at 3) from the optimal side (e.g., median guesses between 8–14 may lead individuals to the optimal equilibrium at 11). More naturalistic framings of coordination allow a wide range of potentially complementary responses, but still emphasize individual payoffs in tasks such as group foraging [12], group path formation [13], and spontaneous traffic lane formation [14]. In all of the aforementioned games, everyone knows the payoff structure and potential goals, and the emphasis is on individual actions and incentives rather than compensatory actions that only allow individuals to succeed if the group succeeds. Matching games do not generally offer individuals separate incentives, but individuals coordinate to the same items or actions by relying on the salience of the options [15], [16]. In contrast, small guesses or reactions in the GBS game can favorably compensate for large guesses or reactions from other members.

Other games emphasize some key elements of the GBS game. For instance, beauty contest games [17] are relevant for their examination of iterated reasoning, and in the GBS game, group members can under-compensate or over-compensate for the guesses that they *think* team members will make. This is akin to the unilateral decisions that adjust how much food one brings to a potluck dinner. The GBS game also complements coordination games geared towards larger populations, such as minority, majority, and business entry games. Minority [18], [19] and majority games [20] lead participants to respectively differentiate and coordinate their strategies, and business entry games occupy a gray area between these extremes [21]. Unlike these games, the GBS game allows both coordination and differentiation of strategies (substitutable or complementary strategies [22]), and it is informative to see which types of strategies are used when members coordinate to an unknown shared goal. Groups could perform best when all members adopt the same strategy, or they could perform best when members use complementary strategies. This article presents an initial investigation of group coordination to a shared goal using the novel GBS task.

## Results

### Group Coordination Results

**Figure 1:**
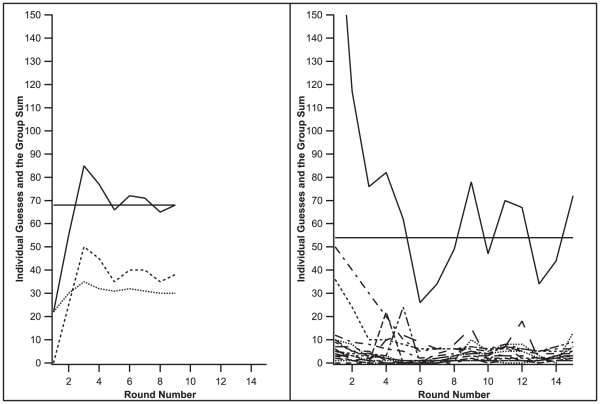
Coordination in one small, 2-person group game (left) and one large, 17-person group game (right) with directional feedback. The solid horizontal line indicates the server's number, and the other solid line indicates the group's sum on a given round. The dashed lines are individual participants' guesses.

We tested a variety of empirical questions regarding the GBS game, including whether groups could successfully coordinate in the task, the effects of increased information (numeric vs. directional feedback), the effects of group size, and the strategies and limitations of coordinating groups. As described in the Methods section, each group completed 10 games, with successive games alternating between numeric and directional feedback from the server. Figure 1 shows directional feedback games from a 2- and 17-participant group, and all game graphs for the 18 groups are available on our website: http://cognitrn.psych.indiana.edu/GBS_graphs.zip.

For many of the analyses, we defined “small groups” as groups with 2 or 3 participants, and “large groups” as groups with 10 or more participants. These group sizes showed strongly contrasting behavior, while the medium-size groups displayed a mixture of behaviors from the two group types. Table 1 reports the average rounds to solution for small, medium, and large groups in numeric and directional feedback games, respectively. A 2 (Feedback: numeric or directional)×3 (Group size: small, medium, or large) mixed groups ANOVA showed main effects for feedback and group size. Numeric feedback games were solved significantly faster than directional feedback games, *F*(1,15) = 24.86, *p*<.001, presumably because the numeric feedback games allow individuals to more precisely modulate their reactions to the group feedback. Small groups solved the games significantly faster than larger groups, *F*(2,15) = 25.07, *p*<.001, and all pairwise comparisons between group sizes were significant. The marginal interaction between feedback type and group size, *F*(2,15) = 3.53, *p* = .055, appears to be driven by small groups' better utilization of numeric feedback compared to the medium and large groups.

**Table 1:** Mean Rounds to Solution (and Standard Deviations) For Different Group Sizes and Feedback Types.

	Numeric feedback	Directional feedback
**Small groups**	4.31 (.82)	6.78 (1.08)
**Medium groups**	8.72 (1.69)	9.78 (1.86)
**Large groups**	11.05 (2.99)	11.95 (1.84)

**Figure 2:**
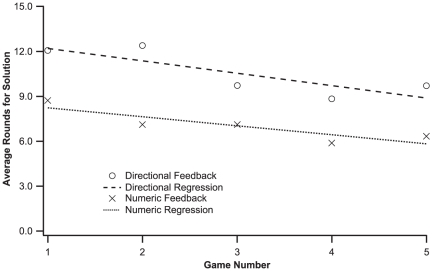
For all groups, the average number of rounds needed for group coordination significantly decreases with group experience, from the first to the fifth directional feedback game, and the first to the fifth numeric feedback game. The dashed lines represent the best-fit. Large and small groups show similar learning trajectories in each condition.

One can imagine large groups allowing individuals' choices to cancel each other out, thus coordinating to the solution more rapidly, but instead the larger groups exhibited larger oscillations, and small groups, with their fewer degrees of freedom and decreased uncertainty, coordinated more quickly. All group sizes showed similar improvement across games, with a −.264 correlation between game number and average rounds to solution, *p*<.001, and both large and small groups showed approximately the same correlations, −.270 and −.273, respectively (the medium size groups slightly lower the average). Figure 2 shows similar improvements with practice in numeric and directional feedback games.

### Adaptive Strategies and Agent-based Models

**Figure 3:**
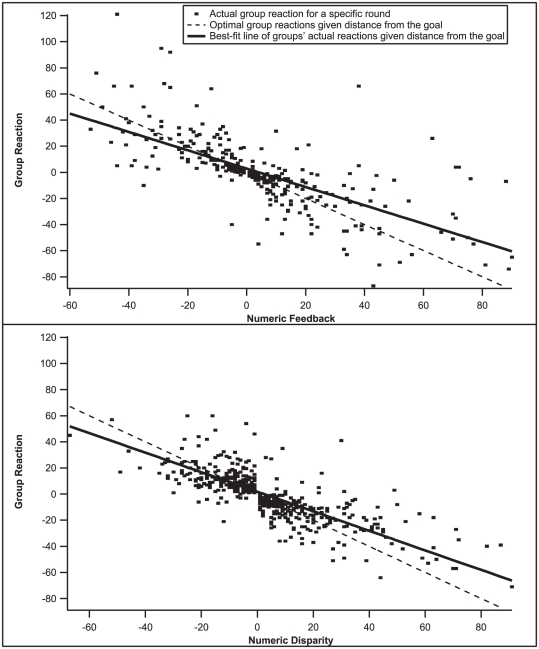
The Y-axis indicates groups' reactions following a given magnitude of numeric feedback on the X-axis (top graph), or an unknown disparity level (the group is only told “Too High” or “Too Low”) in directional feedback games (bottom graph). In both graphs, the solid line is the best-fit line for the data, indicating under-reactivity from the optimal dashed line.

In order to examine the consistency of behaviors among participants, we calculated each participant's “reactivity” according to the formula (G_r_ – G_r-1_) if the group's sum was lower than the target number on the previous round, and (G_r-1_– G_r_) if the group was too high, where G_r_ is the participant's guess on round r. Groups generally underreact, as shown in Figure 3, though only small groups significantly underreact. These results are particularly revealing for directional feedback games, because groups react surprisingly close to the best-fit line despite only receiving directional information. In these cases, individuals may follow a strategy of gradually decreasing their reactivity over rounds. In numeric feedback games, large magnitude feedback tempts individuals in large groups to overreact and form outliers, but overall, the analyses support a nuanced strategy of decreasing reactivity over time in both feedback conditions. The average reactivity of group members per round significantly decreases over the last six rounds prior to solution (This method of aligning rounds allows for greater comparability between numeric and directional games, and small and large groups, given their different solution times), ß = −.326, *p* = .001. However, a paired samples *t* test for all games of all groups reveals that participants significantly decrease their reactivities when the feedback direction changes from one round to the next (mean decrease of 1.55), but maintain approximately the same reactivity (mean decrease of .11) when the feedback direction remains the same, *t*(148) = 4.75, *p*<.001.

Using agent-based models, we tested several reactivity strategies. For each model, we ran 18 groups in 10 directional feedback games, and we matched group sizes to our empirical groups. The source code for all models is available on our website: http://cognitrn.psych.indiana.edu/Main.txt. Each agent first sampled from an empirically derived initial guess distribution that took into account group size, such that there were three derived distributions, for large, small, and medium group sizes. On the second round, agents chose a reactivity from a uniform random distribution with a range of 0 to (50–current guess) if the group was too low on the previous round, and from a range of (−1*current guess) to 0 if the group was too high on the previous round. In order to maintain more realistic reactivities, we further constrained agents to sample until they chose a reactivity within the range −20 to +20. Model 1 and Model 2 agents continued sampling reactivities in this fashion for every round of a game, but Model 2 agents probabilistically decreased their sampled reactivities across rounds. On each round, each possible reactivity number in the range −20 to +20 had a .5 probability of decreasing by an integer chosen from the uniform random range 0 to 5. For example, a Model 2 agent that would have chosen a +18 reactivity in round 6 may actually increase its guess by +12, because the chosen +18 reactivity was decreased across rounds. These random decreases were computed separately for each group game. Models 1 and 2 constitute groups that produce reactions in a feedback-consistent direction, and Model 2 adds the assumption that reactions decrease over time. Models 3 and 4 replace these random reactivity decreases with the notion of agent consistency, Each agent sampled a reactivity on the second round, and on each subsequent round, a Model 3 agent had a .5 probability of decreasing its reactivity by an integer chosen from the uniform random range 0 to 5, while a Model 4 agent only decreased its reactivity when the group feedback changed (e.g. from “Too High” to “Too Low”), and otherwise maintained the same reactivity from round to round. Thus, these models tested whether consistent agents should simply decrease their reactivities over time, or selectively decrease their reactivities when the feedback changed, as our empirical results support.

Model 4 coordinated significantly faster than the other models (means: Model 1 = 13.63, Model 2 = 12.84, Model 3 = 12.00, Model 4 = 10.29, Empirical = 10.51), *p*<.001 for all pairwise model comparisons with Model 4, and was indistinguishable from our empirical results for directional feedback games, *p* = .684. The same model can solve numeric feedback games more quickly by modifying the range of initial agent reactivities according to the numeric feedback. Models 1, 2, and 3 were not significantly different from each other in pairwise comparisons, which illustrates the importance of flexible group coordination. Intuitively, Model 4 agents take large steps towards the goal when they are far away, then decrease their step sizes after passing the goal. In contrast, the approximate simulated annealing strategy [23] from Model 3 does not efficiently span large initial-to-goal distances unless it anneals slowly, but slow annealing results in inefficient oscillations around the goal. We further tested this intuition by comparing Models 3 and 4 on extended games that could go up to 30 rounds, and the influence of unsolved games especially hurt the average solution time for Model 3 (means: Model 3 = 18.99, Model 4 = 14.53, *p*<.001).

When we tried to improve Model 3's performance with alternative values for the probability of reactivity decreases per round and the size of the uniform random range, Model 3 still converged on the target more slowly than Model 4 and our human participants because its agents failed to adjust their reactivities according to feedback. A detailed model comparison supported the importance of such flexible adjustment. We ran Model 3, Model 4, and three mixture models 10,000 times each and computed the likelihood of the empirical data given each model's results. The respective mixture models followed the Model 3 policy 25%, 50%, or 75% of the time and followed the Model 4 policy the rest of the time. All of the mixture models provided significantly better fits for the empirical data than the constrained Model 3 (χ^2^(1) = 6.64, *p*<.01 for the 25/75 mixture model, χ^2^(1) = 10.74, *p*<.01 for the 50/50 mixture model, and χ^2^(1) = 15.52, *p*<.01 for the 75/25 mixture model. However, none of the mixture models provided a significantly better fit for the empirical data than Model 4, and the 25/75 mixture model gave a significantly worse fit than Model 4, χ2(1) = 8.81, p<.01. Flexible adjustment of reactivities based on feedback appears to be a critical aspect of the coordination of empirical groups.

Groups in numeric feedback games clearly do not pursue the expedient normative strategy in which *everyone* adjusts their guesses by 
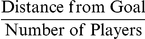
, plus a further increment by 1 with probability 
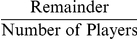
. Our analyses indicate that 26% of numeric feedback rounds were evenly divisible for small groups, compared with 3.2% for large groups, t(11) = 2.50, *p*<.05. However, for these evenly divisible rounds, participants rarely employed the normative strategy, with an average of 14.9% of small group members and 0% of large group members employing the strategy on applicable rounds, t(11) = 1.59, *p* = .14. Instead, in conjunction with our empirical results that participants' reactivities decrease when the group feedback changes, our models suggest that human groups use a flexible, adaptive strategy for group coordination when members are uncertain about others' actions.

### Role Differentiation

**Figure 4:**
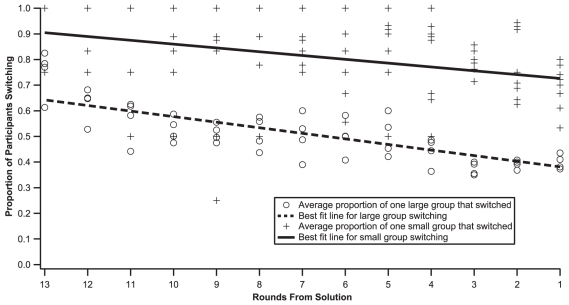
A smaller proportion of group members changes guesses as the group approaches the solution, as measured by rounds before solution on the X-axis. Members of small groups (+s) altered their guesses on successive rounds more often than large groups (circles). The lines are best-fit lines for small and large groups, respectively.

The results so far have implied similar coordination mechanisms in small and large groups, but our final analyses show striking divergent behavior. Groups were clearly able to coordinate to shared goals in the GBS task, but our experiences in real world tasks (e.g. potluck dinners, committees, athletic teams, etc.) suggest that group size has a large effect on coordination. To this end, we calculated the variance of reactivities within individuals (Did a participant exhibit consistent reactivities across rounds?) and between individuals (Did all group members have similar average reactivities?). For each of these analyses, we used groups – rather than individuals – as the unit of analysis by averaging over the individuals within a group. Variance within individuals significantly decreases over rounds (ß = −.519, *p*<.001) for large groups, but marginally increases for small groups (ß = .165, *p* = .083). Similar results (ß = −.164, *p*<.001 for large groups, and ß = .117, *p* = .057 for small groups) are obtained when the results are analyzed at the level of individual participants rather than the group, but such an analysis may not be ideal given the inherent statistical dependencies among members of a group. The variance of reactivities across members of large groups marginally increases over games (ß = .291, *p* = .068), and greater variance among large group members significantly predicts faster coordination (ß = −.395, *p* = .012). In contrast, the variance of reactivities across small group members significantly decreases over games (ß = −.370, *p*<.001), and does not predict solution time. In a more detailed analysis separately examining directional and numeric games, the decreased variance of reactivities across small group members is only significant for directional feedback games (ß = −.377, *p* = .014), while the increased variance across large group members achieves significance for only numeric feedback games (ß = .485, *p* = .041). The average reactivity of large group members also decreases across games (ß = −.313, *p* = .049), but there is no such relationship for small groups (ß = −.04, *p* = .708). Finally, on any given round, a significantly smaller proportion of large than small group members adjust their guesses (Figure 4).

## Discussion

Our results suggest that it is beneficial for members of large groups to differentiate themselves from each other and then maintain those roles in order to foster a predictable environment for subsequent adjustment and coordination. Members of both large groups and small groups react flexibly in the directional feedback games, but small group members do not show such restraint for quickly coordinating in the numeric feedback games. The GBS game is much more difficult for large groups, but increased information leads large group members to develop specialized roles in numeric feedback games. All of the large group members are pursuing a shared goal, but some pursue this by adjusting their guesses, while others adopt small or zero reactivities in order to decrease the group uncertainty. Such inactive individuals are different than free-riders in public goods games. Inactive participants may be intentionally helping the group in the GBS game by decreasing the noise in the system, much like participants in a large group meeting may restrain themselves from talking in order to facilitate a group solution. Our analyses indicate that large groups coordinate more quickly when group members assume these complementary roles. Ironically, allowing altruistic punishment [24], [25] of inactive individuals could actually harm the entire group's performance. Members of committees and group projects may resent individuals who do not contribute sufficiently, but in many cases, increased contributions will delay an integrated final decision or product.

In informal post-task interviews, large groups invariably had many participants who stated that they stopped reacting once the group was close to the goal, because they assumed someone else would react, and having too many reactive people would risk overshooting the target solution. In this respect, the GBS game is a paradigmatic task where *orderly* diffusion of responsibility is a good thing in contrast to its often tragic consequences in situations of social helping [26]. A simple strategy for dropping-out and ceasing to react can lead to deadlock if too many people adopt it. Engaging in higher-order reasoning also runs risks. In each large group, at least one person mentioned attempting to compensate for an anticipated group overreaction by reacting in the opposite direction when the group neared the goal. Analyses indicate that groups would have coordinated faster without this extra compensation.

These findings can be viewed as a preliminary investigation due to the limited number of tested groups. After we had originally prepared this paper, a similar game by Bavelas was brought to our attention [27]; however, the earlier research does not appear to have been published in a primary research outlet, and it is unclear if more than a few groups were tested. The research only included groups of five participants who were explicitly told what the group sum ought to be. Bavelas found that numeric feedback leads to worse group performance than simply indicating that the group is wrong, but we question the replicability of that result given that our groups consistently coordinated more quickly when they received more informative numeric feedback.

Overall, we believe the current work introduces the GBS game as a simple experimental paradigm that can elucidate the mechanisms of group coordination to a *shared* goal that can only be reached together, whereas Battle of the Sexes, order-statistic games, and market entry games offer simple experimental platforms for studying coordination of single actions and maximization of *individual* returns (as well as total collective returns). Role specialization in large groups merits further study within the GBS framework and in ecological studies of real world groups, and future research with the GBS task could examine the limits of coordination and specialization as a function of task difficulty, group history, and the role of individual incentives.

With regards to task difficulty, given our hypothesis that differentiation develops as an adaptation to task difficulty, we expect a lack of differentiation in a modified GBS task that only demands coordination to an interval around the target number (e.g. 65–75 for a target number of 70). Conversely, a more difficult task could promote differentiation even in small groups. An extension of the GBS game could embed participants in multiple overlapping networks, so each participant submits a number to simultaneously reach correct sums in multiple groups. Such a version may resemble the coloring problem [28], where human participants successfully coordinate by dynamically adjusting their color so that no network neighbor shares the same color. The coloring problem requires differentiation among neighbors in order to achieve local coordination, but a similar approach to manipulating network structure may be useful for exploring coordination in different social structures as well as examining adaptive team assembly mechanisms [29] and “downsizing” mechanisms in group coordination.

The influence of group history can be tested by changing group composition after several games. Previous research indicates that diversity [30] and transactive memory systems with divisions of cognitive labor [31], [32] can improve group problem-solving. The benefits of a diverse team often outweigh the benefits of teams culled from best-performing individuals [30]. However, diversity is particularly helpful when group members recognize other members' roles [33], and group members sometimes fail to adapt their roles to changing group conditions [34], [35], which suggests that members of our large or small groups may require significant adjustment periods if we shift group sizes or memberships. Weber has attempted to explain the existence of group coordination by showing that large groups (up to 12 members) can be gradually grown from experienced smaller groups and thereby preserve the good performance in weak-link games [36]. However, our task shows that group coordination can also be explained by members' surprising abilities to specialize their roles while pursuing a shared goal.. These results support recent modeling efforts that show the size and composition of creative groups such as Broadway musical writing teams and scientific research teams evolve to handle task complexity while still minimizing coordination costs [29], but we do not know how quickly the members will adapt to new roles.

Finally, individual incentives may have a detrimental effect different than the one envisioned by weak-link and public goods games. Even when participants are coordinating to a shared goal, too much individual incentive – awarded regardless of collective performance – may impede role specialization and group coordination. For example, a baseball player or corporate executive with a large contract and insufficient team performance incentives may make little effort to coordinate with the team and achieve the shared goal. Future GBS extensions could incorporate individual incentives in addition to the shared goal.

Our initial investigation indicates that the GBS game is a useful framework for testing self-organized division of labor, role development in groups, and relations between individuals' strategies and group-level outcomes. Many real world situations (potluck dinners, academic departments, sports teams, corporate divisions, committees, seminar classes, etc.) intrinsically involve actors adjusting their contributions in order to achieve a mutually satisfactory group goal. These tasks cannot be solved by lone individuals, and the participation of other individuals inevitably brings uncertainty. Our results suggest that teams of individuals with no communication and minimal shared history automatically adjust their roles within their group so that they coordinate appropriately, and these results are particularly surprising given that repeated play could easily establish norms and shared conventions [37], rather than a spontaneous division of labor. Future studies could test a larger number of groups with greater control over the group sizes, and they could examine the influences of task difficulty, group history, and individual incentives on coordination and role specialization.

## Methods

### Participants

Participants were 106 undergraduate students at Indiana University who received course credit for approximately 1 hour of participation. All empirical research was approved by the IRB at Indiana University, and participants were given written informed consent and provided signatures. Participants were run in 18 GBS experimental sessions with the following group sizes: 2, 2, 2, 2, 2, 2, 3, 3, 3, 4, 4, 4, 6, 7, 10, 16, 17, 17. Each group participated in 10 games, alternating between directional feedback games and numeric feedback games. Participants were instructed not to talk to each other, and they were informed that there would be a total of 10 games and they would finish the experiment more quickly if their group quickly coordinated to the solutions. Participants were not otherwise compensated, but in our experience of running the experiments, participants enjoyed successfully coordinating to the solutions. There were audible sighs when the group narrowly missed a goal, and minor celebrations when the group reached a goal. We did not highlight the number of participants in a group, but that information was available, given that all group members were simultaneously present and visible in the computer laboratory.

### Materials and Procedure

Participants sat in a university computer lab at personal computers running the game via client Java applets connected to a computer server. Before each game, the server randomly chose a target number between 51 and 100. During each round, each participant entered a guess between 0 and 50. After a 15 second guessing period elapsed, the server compared the sum of participants' guesses to the group's target number, broadcast the same feedback to all participants' screens, and began the next round. Participants only knew the group sum's relation to the target number (e.g. “Too high” for directional feedback games, or “Too high by 17” for numeric feedback games), without knowing the target number or the current group sum. If the participants' guesses correctly summed to the target number, or if 15 rounds passed unsuccessfully, then the game ended, and the next game began after a short delay.
